# Amifostine Attenuates Doxorubicin-Induced Subacute Hepatic and Renal Toxicity in Rats

**DOI:** 10.3390/ijms27146419

**Published:** 2026-07-19

**Authors:** Vesna Jaćević, Viktorija Dragojević-Simić, Jelica Grujić-Milanović, Silva Dobrić, Dubravko Bokonjić, Zoran Milovanović, Sladjan Milanović, Radoje Simić

**Affiliations:** 1 Department of Experimental Toxicology and Pharmacology, National Poison Control Centre, Military Medical Academy, Crnotravska 17, 11000 Belgrade, Serbia; 2 Department for Pharmacological Science, Medical Faculty of the Military Medical Academy, University of Defence in Belgrade, Crnotravska 17, 11000 Belgrade, Serbia; vdragsim@gmail.com; 3 Centre for Clinical Pharmacology, Military Medical Academy, Crnotravska 17, 11000 Belgrade, Serbia; 4 Department of Cardiovascular Research, Institute for Medical Research, National Institute of the Republic of Serbia, University of Belgrade, 11129 Belgrade, Serbia; jeca@imi.bg.ac.rs; 5 Vojnosanitetski Pregled, Medical Faculty of the Military Medical Academy, University of Defence in Belgrade, Crnotravska 17, 11000 Belgrade, Serbia; silva.dobric@gmail.com; 6 Special Police Unit, Police Department of the City of Belgrade, Ministry of Interior, Trebevićka 12/A, 11030 Belgrade, Serbia; tinahoks41@gmail.com; 7 Department for Biomechanics, Biomedical Engineering and Physics of Complex Systems, Institute for Medical Research, National Institute of the Republic of Serbia, University of Belgrade, 11129 Belgrade, Serbia; sladjan.milanovic@imi.bg.ac.rs; 8 Institute of Mother and Child Health Care of Serbia “Dr Vukan Čupić”, University of Belgrade, Radoja Dakića 6-8, 11000 Belgrade, Serbia; simicradoje@yahoo.com; 9 Medical Faculty, University of Belgrade, dr. Subotića 8, 11000 Belgrade, Serbia

**Keywords:** amifostine, doxorubicin, hepatotoxicity, nephrotoxicity, rats, subacute toxicity

## Abstract

This study aimed to evaluate the general, hepatoprotective and renoprotective effects of amifostine (AMI) in rats treated with a large, single dose of doxorubicin (DOX), assessed at 7 and 56 days after administration. Rats were divided into six experimental groups: Control (0.9% NaCl intraperitoneally (ip)), amifostine (AMI300, 300 mg/kg (ip)), doxorubicin (DOX6, 6 mg/kg (ip)), doxorubicin (DOX10, 10 mg/kg (ip)), doxorubicin (DOX6, 6 mg/kg (ip)) + amifostine (AMI300, 300 mg/kg (ip) 30 min before DOX6), and doxorubicin (DOX10, 10 mg/kg (ip)) + amifostine (AMI300, 300 mg/kg (ip) 30 min before DOX10). Absolute liver and kidney weights were significantly increased in AMI300 + DOX6-treated animals compared to DOX6 only on day 56. In the group of DOX10-treated rats, AMI300 significantly prevented changes in the number of white blood cells during the four weeks after treatment. The severity of hepatic and renal injuries in the DOX6-treated groups was also significantly less in rats pretreated with AMI300 on day 56 (*p* < 0.01). Our results indicate that hepatic and renal protection significantly contribute to the successful use of AMI against DOX-induced subacute toxicity in rats.

## 1. Introduction

Doxorubicin (DOX) is an anthracycline antibiotic derived from *Streptomyces peucetius* bacteria [1]. It is a potent chemotherapy drug used to treat a wide variety of cancers, including breast cancer, lymphomas, sarcomas, ovarian, thyroid, and lung cancers [2,3,4,5]. Although effective, DOX has notable cardiotoxicity and can lead to heart failure [6]. Myelosuppression, mucositis, alopecia, and red-coloured urine are common but reversible side effects of DOX [7]. Doxorubicin acts through multiple mechanisms, such as DNA intercalation and disruption of DNA and RNA synthesis, which prevents cancer cells from dividing properly. It is also considered that DOX blocks the enzyme responsible for unwinding DNA strands, leading to DNA breaks and cell death. Additionally, DOX can inactivate insulin-like growth factor 1 and generate reactive oxygen species (ROS) that damage cellular components, including DNA, proteins, and membranes [8,9,10,11,12]. Numerous experimental models addressing both the therapeutic and toxic effects of DOX exist [13,14,15].

In the liver, DOX is primarily metabolised by aldo-keto reductase (AKR1A) and carbonyl reductases (CBR1/CBR3) into the active metabolite doxorubicinol [16,17]. It can also undergo one-electron reduction to form a semiquinone radical, which generates ROS, superoxide anions (O_2_^−^), hydroxyl radicals (OH^•^), and hydrogen peroxide (H_2_O_2_), leading to hepatotoxicity [18,19]. Peroxidation continues involving the proteins and lipids of the hepatic cell membrane [14,20], resulting in irreversible alterations, such as hepatocyte apoptosis and necrosis [18,21]. This causes structural and functional changes in the hepatic tissue, followed by a marked increase in hepatic enzymes in the blood, primarily alanine aminotransferase (ALT) and aspartate aminotransferase (AST) [22]. Approximately 80% of the total excreted drug amount is eliminated via bile, in which about half of the dose is excreted in the unchanged form, and the rest are conjugates [23]. However, the most significant DOX adverse effects in patients are myelosuppression, cardiotoxicity, and gastrointestinal side effects [1].

Moreover, it has the potential to adversely affect other organs, including nephrotoxicity [24]. The exact mechanism by which DOX causes renal damage remains unclear. It has been confirmed that DOX lowers the levels of major antioxidant enzymes, such as catalase (CAT) and superoxide dismutase (SOD). Oxidative stress caused by DOX overwhelms the natural antioxidant defences of the body and damages cellular components [13,20,25,26]. Some of the studies have shown that another possible mechanism of DOX-induced renal damage is inflammation. In particular, DOX has been shown to increase the production of proinflammatory cytokines such as tumour necrosis factor-α (TNF-α) and interleukin-6 (IL-6) [27,28]. Experimentally, DOX has been shown to cause hyperplasia of the mesangial matrix, collagen deposition, narrowing of the capillary loop, and disruption and detachment of podocyte foot processes [29]. Podocytes affected in this way are unable to adequately perform renal glomerular filtration, disrupting the structure and function of the glomeruli and causing proteinuria, increased serum creatinine (SCr) and blood urea nitrogen (BUN) [30]. In experimental models of proteinuric kidney disease, DOX induces a reliable model of focal segmental glomerulosclerosis, characterised by rapid podocyte injury, severe proteinuria, glomerulosclerosis, and tubular atrophy [31,32]. Progressive loss of renal function is followed by increased levels of SCr, uric acid, and BUN [33].

Amifostine (AMI) is a cysteamine derivative with cytoprotective properties, commonly used to reduce the side effects of chemotherapy and radiation therapy, commonly called the gold standard of cytoprotection [34,35]. It scavenges free radicals and protects healthy tissues due to the action of alkaline phosphatase of the cell plasma membrane, which converts AMI to the free thiol metabolite WR-1065, an active form of the drug [36]. Once inside the cell, AMI scavenges free radicals, donates hydrogen, induces cellular hypoxia, releases glutathione from its bond with cell proteins, and forms mixed disulfides, among other effects. It protects cells from the harmful effects of ionising radiation and various chemotherapeutic agents [37,38].

Our previous research in different experimental rat models treated with single high doses of DOX [13,25], as well as cumulative low doses of DOX [34,39], showed that AMI has a significant bone marrow and cardioprotective effect. We also showed that rats previously treated with AMI had less severe hepatic and renal histological changes caused by cumulative low doses of DOX [39]. All of these studies have shown organ-protective effects of AMI against its toxicity, but AMI efficacy can vary depending on the DOX dose, its administered regimen, and the examined organ systems.

The study aimed to investigate and further elucidate the protective effects of AMI on hepatic and renal toxicity following the application of large single doses of DOX during a two-month period.

## 2. Results

### 2.1. General Health Condition, Body Weight, Liver and Kidney Weight of the Experimental Animals Subjected to Treatments

In animals treated with both doses of DOX, DOX6 and DOX10, there were clear signs of general weakness, adynamia, hair loss, and pronounced hemorrhagic diarrhoea. In the DOX10 group, irritability was clear on the second day after therapy, and all animals in this group died 4 weeks after treatment. Animals from other experimental groups had normal behaviour. The body mass of rats treated with DOX6 and DOX10 was significantly lower in comparison to the control group 7 days after treatment (Figure 1).

On the other hand, seven days after application, the body weight of both groups of rats protected with AMI300 was lower compared to the control group (*p* < 0.05), but significantly higher than in the group that was treated with DOX10 only. During the first week of the study, the total body mass of the animals treated only with AMI300 did not change significantly compared to the control (Figure 1).

In the rest of the study period, the body mass of rats treated with DOX6 only was significantly lower in comparison to the control group, while all animals in the DOX10-only-treated group died 4 weeks after treatment.

The body weight of rats protected with AMI300 was significantly higher than in the group that was treated with DOX6 during the whole study period (*p* < 0.05 and 0.01, respectively). The total body mass of the animals treated with AMI300 did not change significantly compared to the control group during the whole study period (Figure 2).

Seven days and 56 days after treatment, there were no statistical differences between the absolute liver and kidney weights in rats treated only with DOX6 in comparison to the control (Figure 3 and Figure 4). On the other hand, the absolute weight of the liver and kidney of rats that only received DOX10 was significantly lower in comparison to the control during the first four weeks of the study (*p* < 0.01 and *p* < 0.001, respectively). In addition, the absolute liver weight of the rats treated with AMI300 + DOX6 has been statistically different in comparison with DOX6 only at the end of the study period (*p* < 0.01) (Figure 3 and Figure 4).

Moreover, the absolute liver weight of animals treated with DOX6 or DOX10 and pretreated with AMI300 was statistically lower in comparison to the control group of rats only on day 7 of the study (*p* < 0.05) (Figure 3).

### 2.2. Haematological Parameters in the Blood Serum of the Experimental Animals Subjected to Treatments

Application of DOX6 in rats resulted in significant changes in the number of peripheral blood cells. The number of leukocytes significantly increased on day 7 after treatment and remained elevated until the end of the study (Figure 5A). Pretreatment with AMI300 prevented these changes, especially during the first two weeks after administration of DOX6, when the difference was significant (*p* < 0.05). However, in the experimental group of rats that was treated only with DOX10, the number of white blood cells was significantly decreased in the first seven days of treatment in comparison to the starting value (*p* < 0.05). It was followed by an increase, which was significant on day 14 of the study and after that, their gradual decrease until the fourth week after treatment, when all rats died, could be noticed (Figure 5A). The number of leukocytes in the rats treated with AMI300 did not show significant changes from their baseline values.

Application of DOX6 significantly elevated the total number of platelets compared to the basal value. Application of AMI300 before DOX6 successfully prevented these changes in the blood, significantly during the 2 weeks after the treatment, and maintained the basal values of platelets (*p* < 0.05). On the other hand, a decrease of about 50% of the basal level of platelet number was observed during the first week after administration of DOX10 only, with a following increase until the end of the month, when all rats died. However, the total number of platelets in the experimental group that were pretreated with AMI300 was not significantly different from the control animals and was significantly higher than in animals that received DOX10 only during the first 3 weeks after treatment (*p* < 0.05) (Figure 5B).

The negative effect of DOX on red blood cell values was less than that of the leukocyte and platelet values. After the application of DOX6, it did not significantly change the number of red blood cells in the peripheral blood (Figure 5C). A higher number of erythrocytes was observed in animals protected with AMI300 throughout the study period, and a statistical significance was reached on days 21 and 28 after treatments, and after that, gradually decreased below the basal value until the end of the study.

The total number of red blood cells was constantly decreasing after the administration of DOX10, but it was significantly lower than basal values on day 28 of the study, at the last point before the animals died. Animals protected with AMI300 not only survived but also had a higher number of erythrocytes, although not statistically significant.

The effect of DOX on the content of haemoglobin was shown in Figure 5D. Application of DOX10 decreased haemoglobin content in the peripheral blood, which was significantly lower compared to the control group on day 28 of the study. In the group of rats pretreated with AMI300, haemoglobin values remained at the basal level, but their values were significantly higher than in unprotected animals on day 14 after administration of DOX10 (*p* < 0.05).

In the group of rats that received DOX6, a significant increase in hematocrit value was observed on days 28 and 56 after its application in comparison to animals protected with AMI300. Application of a higher dose of DOX decreased the hematocrit level, and its value was significantly lower in comparison to the AMI300 group on day 28 after DOX application. In the group of DOX10-treated rats that were protected with AMI300, a significant decrease in hematocrit value was observed on days 28 and 56 in comparison to animals treated with AMI300 + DOX6.

However, the hematocrit value in the group of animals which have been treated with AMI300 only was not significantly different from that of the control animals during the whole study period (Table 1).

The application of DOX6 or DOX10, with or without AMI300, did not affect the value of mean corpuscular volume, mean corpuscular haemoglobin, or mean corpuscular haemoglobin concentration in the blood of rats in all experimental groups during the whole study period (Table 1).

Also, AMI300 itself has not affected all the examined haematological parameters in comparison to the control group during the whole study period.

### 2.3. Pathohistological Examination of Tissue Alterations of the Experimental Animals Subjected to Treatments

#### 2.3.1. Hepatic Alterations

Microscopic examination of the hepatic tissue sections of the control animals has shown normal histological architecture without changes during the whole study period (Figure 6A and Figure 7A).

On the hepatic sections of the rats treated with AMI300, only mild oedema in the sinusoidal space was seen on days 7 and 56 after its application (Figure 6B and Figure 7B).

On the other hand, hepatic sections of the rats treated with DOX6 only were characterised by numerous intracytoplasmic vacuoles, while the usual radial arrangements of the liver parenchyma were segmentally lost. Moderate oedema and hyperemia were expressed in all sinusoidal spaces. A focal haemorrhage was localised primarily perivascularly and in the periportal space of the hepatic lobule, partly. Hypertrophic Kupffer cells could be seen in the sinusoidal spaces. Almost all hepatocytes were oedematous with marked intracellular degeneration (Figure 6C and Figure 7C). Moreover, irregular cells with marked granularity of the cytoplasm were obtained in the hepatic tissue of rats treated with DOX10 only. In the affected hepatocytes, nuclear pleomorphism with prominent nucleoli was present. In addition, the blood vessels were dilated, with diffuse accumulation of polymorphonuclear cells (PMNCs) (Figure 6D).

The application of AMI300 before DOX6 significantly attenuated the degenerative and vascular changes caused by this chemotherapeutic agent. In this group, the appearance of small, individual vacuoles was observed in a limited number of hepatocytes in the central part of the lobules, while the radial arrangement of the hepatocytes was largely preserved with the presence of mild oedema and hyperemia. Also, the sinusoidal spaces were slightly extended, with an increased number of individual Kupffer cells. Additionally, all blood vessels were just slightly dilated without alterations in their basal membranes and surrounded by individual PMNCs (Figure 6E and Figure 7D).

However, in animals given AMI300 as a pretreatment of DOX10, the AMI300 did not completely succeed in protecting liver tissue against cellular and vascular damage. In this experimental group, the radial arrangement of hepatocytes was lost in the central part of the lobules. The hepatocytes were oedematous and vacuolar degenerated, with small, irregular and hyperchromatic nuclei. Massive oedema and hyperemia were present in the sinusoidal spaces, while focal haemorrhages were localised in the central parts of lobules. All Kupffer cells were hypertrophic and clearly visible in the sinusoidal spaces. The majority of the blood vessels were dilated with discontinuous basal membranes surrounded by an accumulation of PMNCs (Figure 6F and Figure 7E).

#### 2.3.2. Renal Alterations

Microscopic examination of the renal tissue sections of the control animals has shown normal histological architecture without changes (Figure 8A and Figure 9A).

On the renal micrographs of the rats treated with AMI300 only, mild oedema in the renal epithelial cells was seen on days 7 and 56 after treatment (Figure 8B and Figure 9B).

One week after the treatment with DOX6 only, moderate vacuolar changes in renal tubular epithelial cells, extensive vascular changes, and atrophy of some glomeruli were seen (Figure 8C and Figure 9C). Marked thickening of the wall of the juxtaglomerular arteries was associated with loss of some glomeruli in DOX6-treated rats on day 56 after treatment (Figure 9C).

In some tissue sections of the rats treated with DOX10 only, these lesions progressed to degeneration and reduction of tubular epithelial cells. However, in parallel with renal tubular epithelium disappearance, there was a corresponding increase in the representation of interstitial connective tissue. Also, the initial signs of perivascular fibrosis were seen in some tissue samples (Figure 8D).

At the end of the study period, in DOX6-only-treated rats, the majority of the blood vessels were dilated, with discontinuous basal membranes seen on some sections, surrounded by an accumulation of PMNCs both in the renal cortex and the medulla (Figure 9C).

Furthermore, pretreatment with AMI300 decreased the frequency and severity of the described renal alterations in rats treated with DOX6 and DOX10 during the whole study period (Figure 8E,F and Figure 9E).

### 2.4. Semiquantitative Analysis of Tissue Alterations of the Experimental Animals Subjected to Treatments

#### 2.4.1. Quantification of Hepatic Alterations

Amifostine, applied alone, in a single dose of 300 mg/kg (ip), caused mild structural alterations defined with HDS expressed as 0.37 ± 0.51, while HDS of the control animals was 0.25 ± 0.46 throughout the whole study period. The most intensive degenerative and vascular alterations were seen in the groups treated with DOX6 on day 56 (HDS = 4.75 ± 0.46) and DOX10 on day 7 (HDS = 4.00 ± 0.75) (*p* < 0.001 and *p* < 0.01 vs. DOX6 on day 7 (HDS = 1.62 ± 0.51)), respectively (Figure 10).

In the group of animals treated with AMI300 + DOX10, the established score (HDS = 2.75 ± 0.46) was significantly higher than in the group treated with AMI300 + DOX6 on day 7 of the study (HDS = 1.62 ± 0.51, *p* < 0.01). Moreover, in the group of animals treated with DOX10 and pretreated with AMI300, the established score was the same as that in the group treated with AMI300 + DOX6 on day 56 of the study (HDS = 3.00 ± 0.75) (Figure 10).

#### 2.4.2. Quantification of Renal Alterations

When AMI300 was applied alone, in a single dose of 300 mg/kg (ip), a minimal renal alteration was seen (RDS = 0.37 ± 0.51), while in the control group of animals, RDS was 0.25 ± 0.46 during the whole study period. The most intensive renal alterations were observed in rats treated with DOX6 on day 56 (RDS = 4.50 ± 0.53) and DOX10 on day 7 (RDS = 3.75 ± 0.46) (*p* < 0.001 vs. DOX6 on day 7 (HDS = 1.37 ± 0.51)), respectively (Figure 11).

One week after treatment, pretreatment with AMI300 significantly decreased the intensity of the renal alterations caused by DOX10 (RDS = 2.50 ± 0.53) (*p* < 0.01 vs. DOX10 on day 7 (RDS = 3.75 ± 0.46)). However, in the experimental group treated with DOX10 and pretreated with AMI300, there was a significant decrease in RDS (3.62 ± 0.51) in comparison with the group of animals treated with DOX6 only (4.50 ± 0.53) (*p* < 0.001), 56 days after treatment. In addition, a less pronounced score, such as RDS = 2.62 ± 0.51, was established when AMI300 was used in the protection of rats treated with DOX6 (RDS = 4.50 ± 0.53) on day 56 of the study (*p* < 0.01 vs. DOX6 only on day 7) (Figure 11).

## 3. Discussion

Doxorubicin was initially recognised as a highly effective anti-tumour agent, but it has also demonstrated toxic properties depending on the duration and dose of treatment [11,12,14,17,31]. The main mechanism of DOX toxicity involves increased oxidative stress due to elevated free radical generation, often accompanied by cardiomyopathy, nephrotoxicity, hepatotoxicity, myelosuppression, and myopathy [1,8,9,10,40,41,42]. Amifostine is intended to protect normal tissue from chemotherapy-induced damage by being rapidly converted in normal tissues to an active cytoprotective thiol metabolite that scavenges free radicals. It has been shown to reduce haematological and mucosal toxicity [37,38]. Data on the protective properties of AMI specifically against DOX are mixed and less consistent, largely depending on the dose, method of application, and duration of treatment [13,39].

In this experimental study, the rats were monitored for eight weeks after the last administration of DOX. In both experimental groups, treatment with DOX led to notable deterioration in general condition, accompanied by reduced activity, lethargy, loss of body weight, piloerection, poor grooming (rough, unkempt fur), weakness, and fatigue associated with anaemia. Our results indicate that AMI, administered at a dose of 300 mg/kg before both doses of DOX, significantly attenuated the effects of this antineoplastic agent. In fact, at the dose of 10 mg/kg, AMI allowed the survival of all experimental animals. This successful result is consistent with our previous finding, according to which AMI at a fourfold lower dose allowed the survival of 90.4% of animals compared to the group that was treated with DOX-only in a dose of 20 mg/kg (ip), administered in small doses, cumulatively, during 28 days [39].

The significant reduction in body weight, which began on the fourth and fifth day after DOX6 and DOX10 application, respectively, is consistent with the results of Rossi et al. [43]. This trend of body mass reduction in both DOX groups continued over the following eight weeks. After administration of DOX, even at low doses, a significant decrease in body weight was observed compared to the control group [44], due to loss of appetite and extensive lesions in the gastrointestinal tract epithelium that limited food intake and utilisation [45]. Weight loss of rats treated with DOX was less pronounced when they were pretreated with AMI300, similarly to in our previous experiment [39].

Administration of different doses of DOX in rats produced haematological alterations [46]. In our study, the observed decline in total white blood cell count, along with reductions in erythrocyte and platelet parameters after higher doses of DOX, suggests generalised bone marrow suppression. This finding is consistent with the well-established myelosuppressive profile of anthracyclines, which preferentially affect rapidly dividing hematopoietic progenitor cells [47]. The temporal pattern, characterised by an early decrease in haematological parameters followed by gradual recovery, corresponds to the typical cytotoxic nadir observed in rodent chemotherapy models [48,49]. High doses of DOX reduce erythrocyte count, haemoglobin concentration, and hematocrit, indicating the development of moderate anaemia. This effect may result from both impaired erythropoiesis and oxidative damage to circulating erythrocytes. Doxorubicin is known to generate ROS, leading to lipid peroxidation and membrane destabilisation, which may contribute to the shortened erythrocyte lifespan [50]. Similarly, thrombocytopenia observed in treated rats likely reflects suppression of thrombopoietic growth factors within the bone marrow [51]. As far as granulopoetic cell lines are concerned, in our previous experiment, it was hypothesised that DOX actually acts as a strong inhibitor of DNA duplication and transcription, since a delayed type of maturation of the neutrophil granulocytes was observed in comparison to the control animals [39]. In both experimental groups, pre-treatment with AMI significantly attenuated the haematological toxicity induced by DOX. In those rats, protective effects could be detected by more or less expressed preservation of total leukocyte and platelet counts throughout the whole observation period, indicating solid protection of myelopoiesis. Moreover, since both doses of DOX exerted a less toxic effect on erythrocyte parameters, haemoglobin, and hematocrit compared to the other two haematopoietic cell lines, the protective effect of AMI was less pronounced, although it remained evident. The protective effects of AMI are primarily attributed to its active thiol metabolite, which scavenges free radicals and enhances DNA repair in normal tissues [52]. Since AMI is preferentially activated in healthy cells via alkaline phosphatase and accumulates less efficiently in tumour tissue, its cytoprotective action is relatively selective [37,42]. In this context, preservation of peripheral blood counts likely reflects reduced oxidative damage to haematopoietic stem and progenitor cells, as well as improved bone marrow cellular integrity [39,53]. AMI reduced the severity of myelosuppression, but did not completely prevent hematologic alterations, indicating that high-dose DOX exposure may exceed the protective capacity of animals. Other authors also indicated that a single high dose of DOX administered in rats induced prominent oxidative stress, which was characterised by high MDA and carbonyl protein, and depression in some of the antioxidant enzymes, both in erythrocytes and in plasma [54]. In our study, AMI obviously reduced oxidative damage to erythrocytes, a well-known effect both in experimental animals and humans for many years [54,55,56]. Additionally, our findings from previous experiments are in accordance with in vitro studies, indicating AMI “cytokine-like” stimulation of the bone marrow cells contributing to its broad-spectrum myeloprotection [37,39]. By attenuating leukopenia, anaemia, and thrombocytopenia, AMI may reduce the risk of infection, bleeding complications, and treatment interruptions in chemotherapy settings, also explaining the better general condition of our protected animals, as well as their reduced mortality.

In our study, relative liver weight varied, increasing or decreasing after DOX treatment depending on the dosing intervals, as shown in other animal studies [37,57]. Some studies show a trend toward an increased liver-to-body weight ratio in DOX-treated groups compared with controls [44]. However, other studies report no significant change in the liver weight ratio, even though biochemical markers such as AST and ALT indicate liver injury [6]. Therefore, our experiment indicated that DOX10 only significantly decreased absolute liver weight, suggesting hepatotoxic effects of DOX. Pretreatment with AMI in both groups (AMI300 + DOX6 and AMI300 + DOX10, respectively) markedly attenuated these changes, but only on day 56 after treatment, indicating a protective effect against DOX-induced hepatic toxicity, which is consistent with the findings of the study by Jahnukainen et al. [35].

Moreover, structural alterations were observed in liver tissue after administration of DOX at both doses (DOX6 or DOX10). In this experimental study, the lower dose of DOX6 induced focal intracytoplasmic vacuolar degeneration, haemorrhage in the perivascular area, with moderate oedema and hyperaemia (HDS = 4.75 ± 0.46 on day 56). In the liver tissue of rats treated with DOX10 alone, blood vessels were dilated, with diffuse accumulation of PMNCs. In addition, hepatocytes were irregularly shaped, with pronounced cytoplasmic granularity and nuclear pleomorphism with prominent nucleoli (HDS = 4.00 ± 0.75 on day 7).

On the other hand, pretreatment with AMI300 significantly attenuates the degenerative and vascular changes caused by this chemotherapeutic agent. In the group treated with AMI300 + DOX6, focal degenerative and vascular changes were observed only in the central part of the lobules (HDS = 3.00 ± 0.75) on day 56 of the study. However, in animals given AMI300 as a pretreatment of DOX10, the AMI300 did not completely protect liver tissue against degenerative and vascular damage, and the radial arrangement of hepatocytes was lost in the central part of the lobules. Moreover, in this group of animals on day 7, the established score was the same as that of the group treated with AMI300 + DOX6 on day 56 of the study (HDS = 3.00 ± 0.75).

Hepatotoxicity induced by DOX has been widely attributed to several interconnected mechanisms, primarily involving oxidative stress, mitochondrial dysfunction, and inflammatory responses in hepatocytes [12,19,34,39]. Previous studies have shown that, following administration, DOX undergoes an enzymatic redox cycle in liver cells, generating excessive ROS such as O_2_^−^, OH, and H_2_O_2_ [10]. The overproduction of ROS overwhelms the endogenous antioxidant defence system, resulting in protein oxidation and lipid peroxidation of cellular membranes [58,59]. Following oral and (ip) administration, DOX accumulates within mitochondria, where it disrupts the electron transport chain, leading to impaired ATP production, loss of mitochondrial membrane potential, and further ROS generation [60]. These alterations trigger mitochondrial permeability transition and release pro-apoptotic factors such as caspase-3 and Bax, ultimately activating caspase-dependent apoptotic pathways [61]. Furthermore, DOX has been shown to stimulate inflammatory signalling through activation of nuclear transcription factors and the subsequent release of pro-inflammatory cytokines, which contribute to hepatocyte apoptosis and necrosis [62].

In this context, cytoprotective agents such as AMI have attracted attention for their ability to attenuate DOX-induced toxicity [34,36,39]. Amifostine reduces liver injury caused by chemotherapy, radiation, or oxidative stress [37]. Its active metabolite WR-1065 acts as a potent free-radical scavenger and enhances endogenous antioxidant defences [51]. Through these mechanisms, AMI can scavenge ROS, reduce oxidative stress in hepatocytes, and ultimately protect liver cells from free radical damage, thereby limiting DOX-induced hepatocellular damage [39,63].

In several studies, depending on the experimental model, DOX treatment may decrease kidney weight or leave it unchanged [53]. Changes in kidney weight are often less pronounced than changes in biochemical markers such as creatinine or proteinuria [64]. Some experiments report no significant changes in kidney weight despite evidence of functional kidney damage [65]. Thus, in our experiment, depending on the doses and length of period after DOX administration, the absolute kidney weight remained unchanged in the DOX6-treated group during the whole study period, while in the DOX10 experimental group, it was significantly lower in comparison to the control group. However, in the group of animals pretreated with AMI300 before DOX6, there was a significant increase in absolute kidney weight compared to the group of animals treated with DOX6 only, as in other studies [35].

In both experimental groups, DOX (DOX6 or DOX10) induced histopathological alterations in renal tissue. Treatment with DOX6 alone resulted in mild tubular epithelial degeneration, associated with interstitial inflammatory cell infiltration, while the higher dose in the DOX10-treated group led to changes, such as focal necrosis, glomerular sclerosis and atrophy. Quantitatively, the most intensive renal alterations were observed in rats treated with DOX6 on day 56 (RDS = 4.50 ± 0.53) and DOX10 on day 7 (RDS = 3.75 ± 0.46), respectively.

On the other hand, pretreatment with AMI300 significantly decreased the intensity of the renal cortical and medullary degeneration, inflammatory cell infiltration and focal haemorrhages caused by DOX10 (RDS = 2.50 ± 0.53) in comparison to DOX10 on day 7 (RDS = 3.75 (0.46)). However, in the same pretreated group of rats, there was a significant decrease in established score (RDS = 3.62 ± 0.51) in comparison with the group of animals treated with DOX6 only on day 56 (RDS = 4.50 ± 0.53).

Administration of DOX is known to produce characteristic histopathological alterations in renal tissue, primarily affecting the glomeruli and renal tubules [30]. Numerous studies have shown that DOX-induced nephrotoxicity is primarily associated with oxidative stress, inflammation, and apoptosis in renal tissues [31]. As an anthracycline chemotherapeutic agent, DOX undergoes redox cycling that promotes the generation of ROS, leading to lipid peroxidation, mitochondrial dysfunction, and structural damage in renal tubular epithelial cells [66]. This alteration is often accompanied by elevated serum creatinine and urea levels [7].

In addition, DOX activates inflammatory pathways and apoptotic signalling cascades, further worsening renal damage. In the pathology of DOX-induced kidney inflammation, nuclear factor-κB (NF-kB) functions as a key transcriptional activator that regulates the expression of various inflammatory factors [67]. Structural changes reflect oxidative stress-mediated injury, mitochondrial dysfunction, and apoptotic processes in renal cells. Through DOX, NF-kB activates proinflammatory cytokines (TNF-α, IL-6, IL-1β) and inflammatory mediators (iNOS and COX-2). During inflammation, activation and phosphorylation of IkappaB kinases promote the phosphorylation of NF-kB, leading to an irreversible inflammatory response [68]. Along with NF-kB, MAPK family proteins are key regulators of inflammation, cell differentiation, proliferation, and ultimately apoptosis [69]. Other studies have confirmed that p38-MAPK activation induces the release of cytochrome C from mitochondria [51]. After cytochrome-C is released, the pro-apoptotic marker BAX increases, the anti-apoptotic marker Bcl2 decreases, and caspase-3 is activated, inevitably leading to renal apoptosis [69].

Amifostine, once it gets into the cell, as active metabolite WR-1065, is able to scavenge free radicals, donate hydrogen, induce cellular hypoxia, release glutathione from its bond with cell proteins, and form mixed disulfides, among other effects [36,37,38]. During our whole experiment, AMI, when applied alone, in a single dose of 300 mg/kg (ip), caused just minimal renal and hepatic alterations, not statistically different from the control group of animals. In our previous study, AMI reduced carrageenan-induced rat paw oedema, achieving a high degree of anti-inflammatory activity, being the most effective at the highest dose tested, and it was actually 300 mg/kg (ip) [25]. The acute local inflammatory response induced by carrageenan is related to neutrophil infiltration and the production of prostaglandins. In our present experiment, diffuse accumulation of PMNCs around dilated blood vessels was seen both in renal and hepatic tissue in rats treated with both doses of DOX, while in AMI-pretreated animals, focal infiltrates or individual PMNCs can be seen. It was shown that carrageenan activated PMNCs and macrophages and released ROS, nitric oxide and other free radicals, and some cytokines, such as IL-6 and Tumour Necrosis Fractor-alpha (TNF-α) [70]. Since it was suggested that ROS, in turn, activate inflammatory cells that lead to the progression of inflammation, it was supposed that a strategy for reducing the ROS could contribute to decreasing the development of inflammation [23,71], which was substantiated in our experiments with AMI [25]. Its antioxidative activity was shown in an in vitro study by using a chemical system demonstrating that WR-1065 scavenged O_2_^−^ and OH^•^, including DOX-derived O_2_^−^ generated by NADH respiration of heart mitochondria particles [72]. Moreover, related to NF-kB, which functions as a key transcriptional activator that regulates the expression of various inflammatory factors, it was shown that AMI exerts a potent inhibitory effect on lipopolysaccharide (LPS)-induced NF-κB activation [73]. Under inflammatory conditions, LPS activates NF-κB via MAP kinases (such as p38) and protein kinase C pathways in a redox-sensitive manner. Amifostine acts by dramatically reducing oxidative stress and suppressing inflammatory hyperactivation of NF-κB, preventing vascular permeability and lung injury in the rodent experimental model [73].

The antioxidative properties of AMI (200 mg/kg) were also demonstrated in a previous study by reducing the expression of 8-hydroxy-2-deoxyguanosine (8-OHdG) and caspase-3 [74]. The formation of 8-OHdG represents one of the most common oxidative lesions in DNA and serves as a sensitive biomarker of ROS-mediated damage in renal tubular epithelial cells [75,76]. Increased 8-OHdG immunoreactivity in kidney tissue, therefore, reflects pronounced oxidative stress and impaired DNA integrity following DOX exposure [77]. High-dose and long-term DOX therapy induces persistent oxidative DNA damage, which can subsequently trigger apoptotic signalling pathways, particularly through mitochondrial injury and caspase activation. Among these, caspase-3 acts as the principal executioner enzyme responsible for DNA fragmentation, cytoskeleton degradation, and programmed cell death in renal cells [78]. Administration of the cytoprotective agent AMI reduced caspase-3 expression and diminished apoptotic loss of renal tubular cells and testicular cells [79,80,81].

## 4. Materials and Methods

### 4.1. Chemicals

DOX (Adriablastina^®^) for (ip) administration was purchased from Pharmacia & Upjohn (Milan, Italy). Amifostine was synthesised in the Chemical Department of the Military Technical Institute, Belgrade, Serbia, by the original procedure previously described [34]. It was prepared for administration by dissolving the substance in a sterilised and apyrogenic 0.9% NaCl solution, ex tempore.

### 4.2. Experimental Animals

Experiments were performed on male Wistar rats (RGD ID: 645122512), 6–8 weeks old (200 to 220 g), bred at the Department for Experimental Animals, Military Medical Academy, Belgrade, Serbia. The experimental animals were housed in plastic cages (Macrolon^®^ cage type 4, Bioscape, Otterndorf, Germany) with sawdust bedding (Versele-Laga, Deinze, Belgium) certified as having contaminant levels below toxic concentrations. The environmental conditions were controlled and monitored by a central computer-assisted system with a temperature of 22 ± 2 °C, relative humidity of 55 ± 15%, 15–20 air changes/h, and artificial lighting of approximately 220 lux (12 h light/dark cycle). The experimental animals had free access to food, commercial pellets for rats (Veterinarski Zavod Subotica, Subotica, Serbia), and tap water from municipal mains, filtered through a 1.0 µm filter (Skala Green, Belgrade, Serbia).

All the above environmental conditions, as well as all the procedures adopted for housing and handling of experimental animals, were in strict compliance with the Guidelines for Laboratory Animal Welfare of the Ethics Committee for Experiments on Animals of the Military Medical Academy, Belgrade, Serbia, which were adopted and are in complete accordance with the current Guidelines for Animal Welfare approved by the European Commission.

The study protocol was approved by the Ethics Committee for Experiments on Animals issued by the Military Medical Academy, Belgrade, Serbia (approved study protocol no.: 282-12/2002; date of approval: 2 December 2002).

### 4.3. Experimental Design

Wistar rats were randomly divided into six experimental groups, each containing sixteen individuals (n = 16). The animals received the following treatments: (1) Control (0.9% NaCl), (2) amifostine (AMI300, 300 mg/kg (ip)) 20 min before saline (1 mL/kg (ip)), (3) doxorubicin (DOX6, 6 mg/kg (ip)), (4) doxorubicin (DOX10, 10 mg/kg (ip)), (5) doxorubicin (DOX6, 6 mg/kg (ip)) + amifostine (AMI300, 300 mg/kg (ip) 30 min before DOX6), and (6) doxorubicine (DOX10, 10 mg/kg (ip)) + amifostine (AMI300, 300 mg/kg (ip) 30 min before DOX10). The eight animals from the DOX10-treated group were sacrificed on day 7, and the remaining eight rats died four weeks after treatment. All animals in the other experimental groups survived until days 7 and 56, respectively. At those time points, eight animals from each experimental group were sacrificed.

### 4.4. General Health Condition and Weight Changes

General health conditions were monitored daily throughout the whole experimental period. The mean values of rat body weight were calculated for each treatment group separately every day during the first week, and once a week for the next 7 weeks, until the end of the experiment. The mean values of liver and kidney weight were calculated for each treatment group separately on days 7 and 56 of the study.

### 4.5. Haematological Assays

On the day of blood collection, all the rats, which fasted overnight, were deeply anaesthetised by 25% urethane (4 mL/kg) (Sigma-Aldrich, St. Louis, MO, USA), immobilised in a ventral position and allowed to breathe spontaneously. Blood samples were collected aseptically from the tail vein, with a needle and plastic syringe, separately from each rat on days 7, 14, 21, 28 and 56 of the study. After collection, a single blood sample was distributed (1 mL aliquots) into a previously designated tube containing 1.5 mg Na_2_EDTA (disodium ethylenediaminetetraacetic acid) (Sigma-Aldrich, Merck KGaA, Darmstadt, Germany). This was well mixed by gentle inversion for complete blood count (CBC) analysis. All blood samples were processed within 4 h and subjected to routine analysis using a haematology auto analyser ERP-9 (Ortho Diagnostic System GmbH, Raritan, NJ, USA).

Standard haematological parameters measured and calculated were: red blood cells (RBC), haemoglobin (HB), haematocrit (HCT), mean corpuscular volume (MCV), mean corpuscular haemoglobin (MCH), mean corpuscular haemoglobin concentration (MCHC), platelets (PLT), and white blood cells (WBC).

### 4.6. Pathohistological Analysis

In order to evaluate the hepatoprotective and renaloprotective effects of AMI300, the animals were sacrificed 7 and 56 days after receiving treatment. Before sacrificing the rats, they were anaesthetised with 25% urethane (4 mL/kg) (Sigma-Aldrich, Merck KGaA, Darmstadt, Germany), immobilised in a dorsal position and allowed to breathe spontaneously. At necropsy, the dissected liver and kidney tissue were carefully spread over a metal tray coated with wax and fixed with 10% neutral buffered formalin solution. Five to seven days after fixation, all tissues were divided into 6 portions in order to be prepared for making sections. After the process of fixation, all tissue portions were dehydrated in graded alcohol (100%, 96% and 70%), xylol and embedded in paraffin blocks. Finally, 2-μm thick paraffin sections were stained by the haematoxylin and eosin (H&E) method.

### 4.7. Semiquantitative Pathohistological Analysis

The type, degree and severity of hepatic and renal lesions, along with the degree of inflammatory cellular infiltration, were assessed in all tissue sections from each animal, and they were counted in separate visual fields at 400× magnification (Olympus BX 43, Olympus, Tokyo, Japan). The severity of hepatic and renal lesions, consisting of oedema, cellular infiltration, haemorrhages, vacuolar degeneration, necrosis, and the distribution of lesions (e.g., focal, multifocal, locally extensive, or diffuse), was assessed and graded by the two independent pathologists. In each section, entire visual fields were analysed using a light microscope according to the previously described 5-point semiquantitative scales [26,39,41]. A severity grade was expressed as hepatic damage score (HDS) and renal damage score (RDS), and the exact method of calculation is shown in Figure 10 and Figure 11, respectively.

### 4.8. Statistical Analysis

Statistical evaluation was performed using commercial statistical software (Stat for Windows, R.7, Stat Soft, Inc., Tulsa, OK, USA, 2008). In the case of continuous data, variables were presented as mean value ± standard deviation (SD). The Kolmogorov–Smirnov test was used for the evaluation of the normality of the presented data. If data were normally distributed, 1-way ANOVA and post hoc Bonferroni tests were used. If data were not normally distributed, Kruskal–Wallis 1-way ANOVA and post hoc Mann–Whitney U tests were used. The differences with values of *p* < 0.05, *p* < 0.01 and *p* < 0.001 were considered significant.

## 5. Conclusions

Based on the present results, AMI administered 30 min before DOX, at a dose of 300 mg/kg, significantly attenuated its effects at both doses (DOX6, DOX10). Animal survival, whole body weight, liver and kidney weight changes, peripheral blood cell count deterioration, and structural changes in the liver and kidneys caused by DOX were prominently improved.

Overall, pathohistological findings indicate that AMI administration reduces the severity of hepatic and renal lesions associated with DOX-induced hepatotoxicity and nephropathy, preserving hepatocytes, as well as tubular and glomerular structures, and contributing to improved hepatic and renal tissue integrity. Therefore, the observed improvement in all parameters following AMI300 administration likely reflects its antioxidant, anti-inflammatory, and anti-apoptotic properties, supporting its potential role as a protective agent against DOX -induced hepatotoxicity and nephrotoxicity.

## Figures and Tables

**Figure 1:**
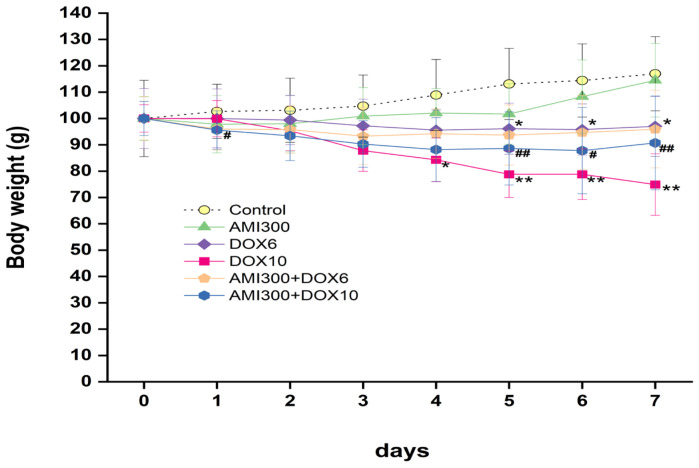
Influence of treatment with amifostine and/or doxorubicin on the body weight of rats during 7 days after treatment. Statistical analysis was performed using the Student *t*-test. The results are expressed as a percentage related to the control group on day 0. *, **—*p* < 0.05, 0.01 for the results in comparison with the control group. #, ##—*p* < 0.05, 0.01 for the results in comparison with the related DOX group. Note: all animals in each experimental group survived until day 7, when 8 animals from each group were sacrificed.

**Figure 2:**
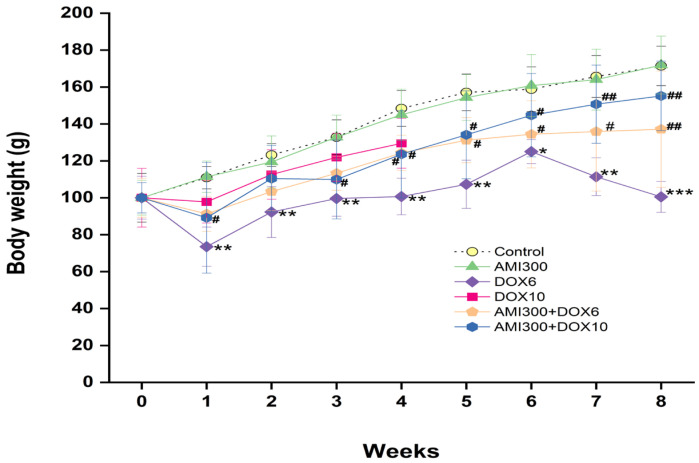
Influence of treatment with amifostine and/or doxorubicin on the body weight of rats during 8 weeks after treatment. Statistical analysis was performed using the Student *t*-test. The results are expressed as a percentage related to the control group on day 0. *, **, ***—*p* < 0.05, 0.01, 0.001 for the results compared to the control group. #, ##—*p* < 0.05, 0.01 for the results in comparison to the DOX6 group. Note: all animals in the DOX10-treated group died 4 weeks after treatment. All animals in the other experimental groups survived until day 56, when 8 animals from each group were sacrificed.

**Figure 3:**
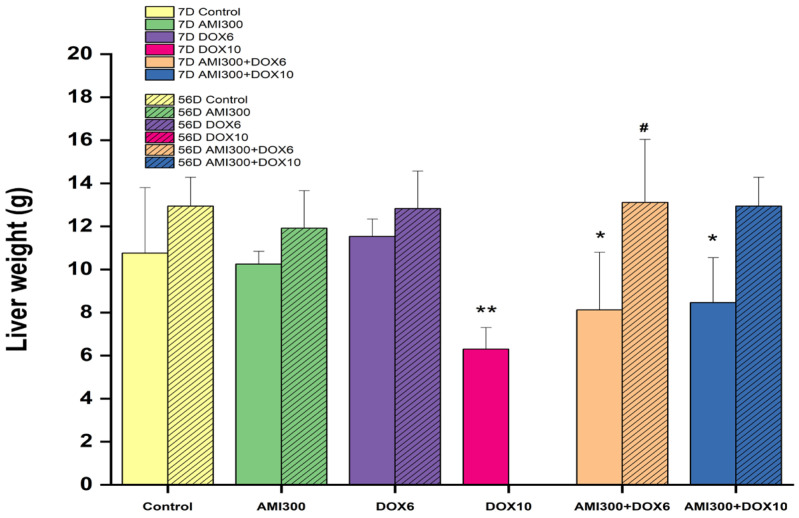
Influence of treatment with amifostine and/or doxorubicin on absolute (g) liver weight of rats during 8 weeks after treatment. Statistical analysis was performed using the Student *t*-test. *, **—*p* < 0.05, *p* < 0.01 in comparison to the control group. #—*p* < 0.05 for the results in comparison with the DOX6 group. Note: all animals in the DOX10-treated group died 4 weeks after treatment. All animals in the other experimental groups survived until days 7 and 56, when 8 animals from each group were sacrificed.

**Figure 4:**
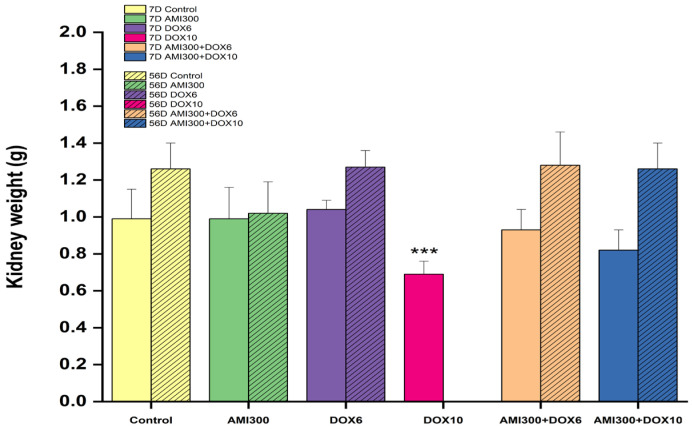
Influence of treatment with amifostine and/or doxorubicin on absolute (g) kidney weight of rats during 8 weeks after treatment. Statistical analysis was performed using the Student *t*-test. ***—*p* < 0.001 for DOX10 in comparison to the control group. Note: all animals in the DOX10-treated group died 4 weeks after treatment. All animals in the other experimental groups survived until days 7 and 56, when 8 animals from each group were sacrificed.

**Figure 5:**
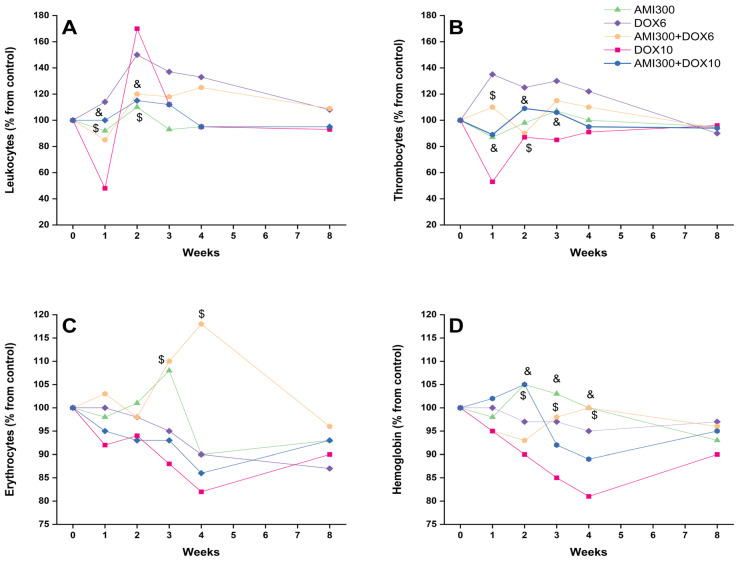
(**A**–**D**) The influence of treatment with amifostine and/or doxorubicin on white blood cells (WBC), platelets (PLT), red blood cells (RBC) and haemoglobin (HB) values during 8 weeks after treatments. Statistical analysis was performed using the Student *t*-test. The results are expressed as a percentage related to the baseline values on day 0. $—*p* < 0.05 in the comparison to the DOX6 group. &—*p* < 0.05 in the comparison to the DOX10 group. Note: all animals in the DOX10-treated group died 4 weeks after treatment. All animals in the other experimental groups survived until day 56, when 8 animals from each group were sacrificed.

**Figure 6:**
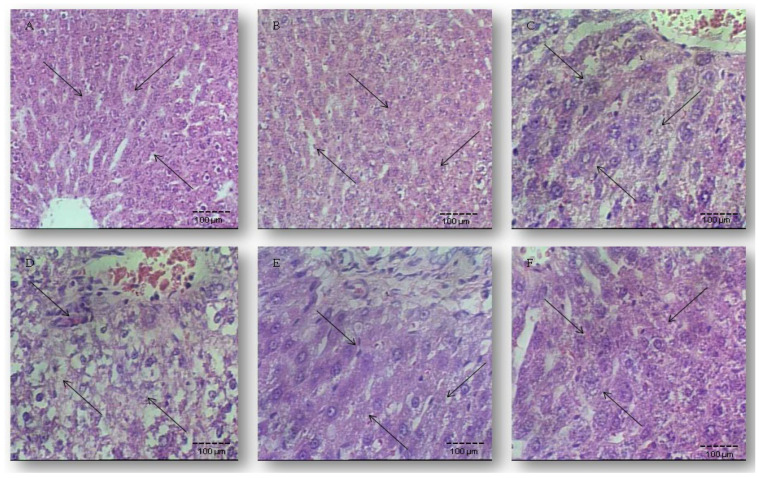
Light micrographs of the hepatic lesions in rats on day 7 after treatment. H&E stain. Magnification 200×. (**A**) Normal histological structure of the hepatic tissue. (**B**) The AMI300-treated group is characterised only by mild cytoplasmic vacuolisation of the hepatocytes and oedema in the sinusoidal spaces. (**C**) In the DOX6-treated group, foci of necrotic cells are surrounded by PMNCs. (**D**) The DOX10-treated group is presented with complete multilobular necrosis and massive accumulation of inflammatory cells. (**E**) In the AMI300 + DOX6-treated group, mild vacuolar degeneration of a single hepatocyte and discrete focal PMNCs infiltrates can be observed. (**F**) The AMI300 + DOX10-treated group is characterised by the granularity of the cytoplasm and nuclear pleomorphism.

**Figure 7:**
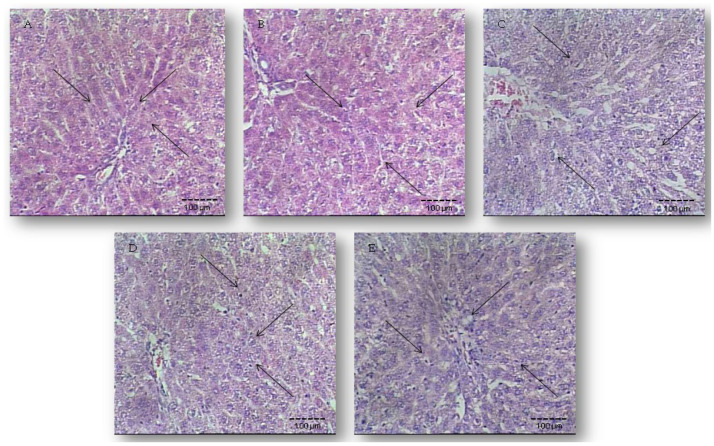
Light micrographs of the hepatic lesions in rats on day 56 after treatment. H&E stain. Magnification 200×. (**A**) Normal histological structure of the hepatic tissue. (**B**) The AMI300-treated group only, mild hepatic vacuolisation and oedema in the sinusoidal spaces. (**C**) The DOX6-treated group, necrotic hepatocytes surrounded with PMNCs and hypertrophic Kupffer’s cells. (**D**) The AMI300 + DOX6-treated group, vacuolarly degenerated hepatocytes with small, irregular and hyperchromatic nuclei. (**E**) The AMI300 + DOX10-treated group, small vacuolar degeneration of hepatocytes and nuclear pleomorphism. Note: all animals in the DOX10-treated group died 4 weeks after treatment.

**Figure 8:**
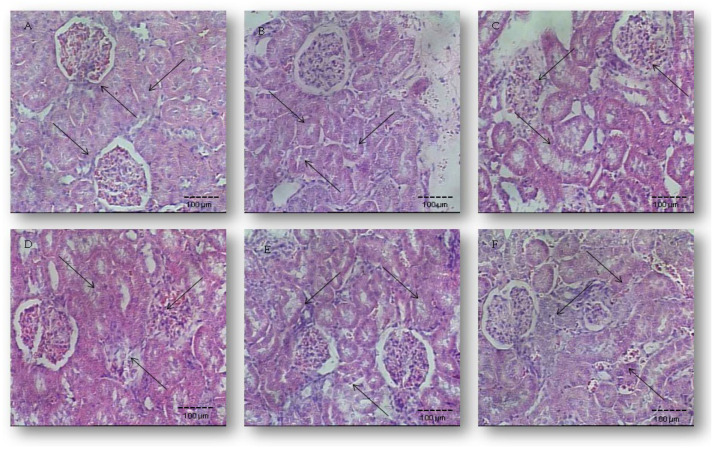
Light micrographs of the renal lesions of rats on day 7 after treatment. H&E stain. Magnification 200×. (**A**) Normal histological structure of the renal tissue. (**B**) The AMI300-only-treated group had mild oedema of tubular epithelial cells. (**C**) The DOX6-treated group showed moderate atrophy of some glomeruli and haemorrhagic foci. (**D**) The DOX10-treated group showed severe degeneration and reduction of tubular epithelial cells. (**E**) The AMI300 + DOX6-treated group showed focal cortical and medullary cell degeneration. (**F**) The AMI300 + DOX10-treated group showed diffuse cortical and medullary cell degeneration surrounded by inflammatory cell infiltration and focal haemorrhages.

**Figure 9:**
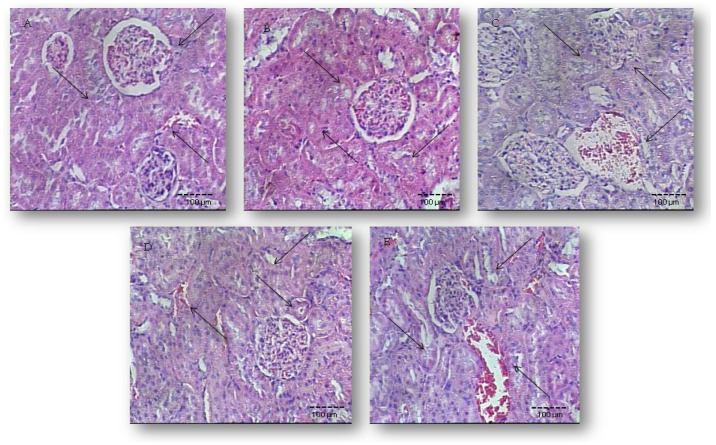
Light micrographs of the renal lesions of rats on day 56 after treatment. H&E stain. Magnification 200×. (**A**) Normal histological structure of the renal tissue. (**B**) The AMI300-only-treated group, with mild oedema of tubular epithelial cells. (**C**) The DOX6-treated group showed atrophy of some glomeruli and haemorrhagic foci surrounded by PMNCs. (**D**) The AMI300 + DOX6-treated group showed mild degeneration and reduction of tubular epithelial cells. (**E**) The AMI300 + DOX10-treated group showed marked degeneration of tubular epithelial cells surrounded by haemorrhagic foci. Note: all animals in the DOX10-treated group died 4 weeks after treatment.

**Figure 10:**
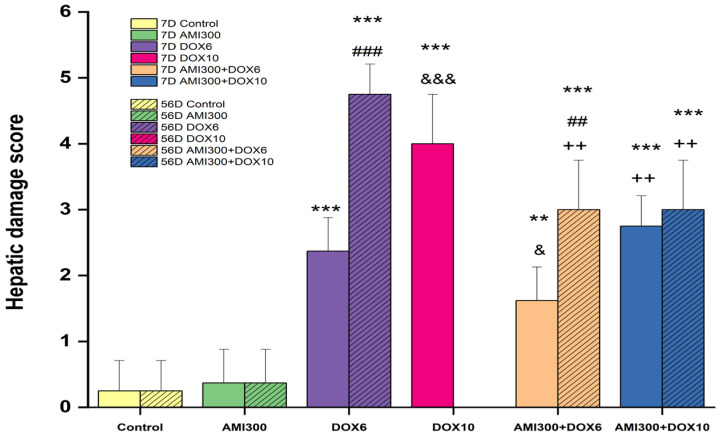
Hepatic damage score—HDS (Influence of treatment with amifostine and/or doxorubicin on frequency and severity of the hepatic lesions in rats). Statistical evaluation was performed using the Kruskal–Wallis test. **, ***—*p* < 0.01, 0.001 vs. control (7th, 56th day), ##, ###—*p* < 0.01, 0.001—vs. the same treatment (7th day), &, &&&—*p* < 0.05, 0.001 vs. DOX6 (7th day), ++—*p* < 0.01 vs. AMI300 + DOX6 (7th day). Note: all animals in the DOX10-treated group died 4 weeks after treatment. All animals in the other experimental groups survived until days 7 and 56, when 8 animals from each group were sacrificed.

**Figure 11:**
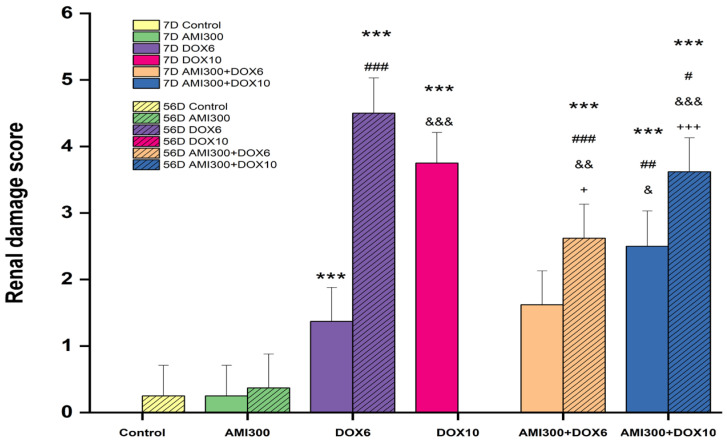
Renal damage score—RDS (Influence of treatment with amifostine and/or doxorubicin on frequency and severity of the renal lesions in rats). Statistical evaluation was performed using the Kruskal–Wallis test. ***—*p* < 0.001 vs. control (7th, 56th day), #, ##, ###—*p* < 0.05, 0.01, 0.001—vs. the same treatment (7th day), &, &&, &&&—*p* < 0.05, 0.01, 0.001 vs. DOX6 (7th day), +, +++—*p* < 0.05, 0.001 vs. AMI300 + DOX6 (7th day). Note: all animals in the DOX10-treated group died 4 weeks after treatment. All animals in the other experimental groups survived until days 7 and 56, when 8 animals from each group were sacrificed.

**Table 1:** Influence of treatment with amifostine and/or doxorubicin on haematocrit (HCT), mean corpuscular volume (MCV), mean corpuscular haemoglobin (MCH), and mean corpuscular haemoglobin concentration (MCHC) values during 8 weeks after treatment.

Treatment (mg/kg)	Days	% ($\bar{\times }$ ± SD)			
		HCT	MCV	MCH	MCHC
AMI300	7	107.30 ± 11.81	101.28 ± 1.81	98.14 ± 1.34	94.85 ± 1.32
	14	101.30 ± 10.23	94.88 ± 1.58	101.33 ± 1.34	107.38 ± 3.00
	21	96.66 ± 8.04	91.46 ± 1.48	103.55 ± 2.69	106.96 ± 4.39
	28	98.27 ± 7.80	95.26 ± 2.34	98.27 ± 7.80	95.26 ± 2.34
	56	102.39 ± 10.23	98.49 ± 1.98	102.39 ± 10.23	98.49 ± 1.98
DOX6	7	107.33 ± 10.20	103.13 ± 7.56	100.52 ± 9.30	97.22 ± 3.97
	14	101.68 ± 11.63	98.96 ± 8.64	99.57 ± 7.23	101.79 ± 6.63
	21	104.35 ± 16.26	96.59 ± 8.50	99.77 ± 7.94	99.59 ± 11.75
	28	111.11 ± 14.59 **	105.69 ± 7.86	105.98 ± 9.24	95.67 ± 12.06
	56	118.65 ± 37.53 **	128.71 ± 7.34	121.76 ± 25.23	91.90 ± 14.58
DOX10	7	87.99 ± 14.62	99.8 ± 5.92	117.08 ± 35.93	118.54 ± 32.46
	14	90.87 ± 12.48	97.84 ± 5.77	96.95 ± 6.85	100.25 ± 3.38
	21	90.82 ± 18.76	107.31 ± 4.36	98.97 ± 9.91	94.18 ± 7.98
	28	78.55 ± 15.32 ***	93.91 ± 7.58	105.89 ± 22.13	114.08 ± 18.21
	56	-	-	-	-
AMI300 + DOX6	7	102.91 ± 12.84	101.31 ± 5.41	91.11 ± 7.29	92.97 ± 4.77
	14	98.74 ± 10.23	101.62 ± 5.02	92.89 ± 6.18	94.77 ± 5.56
	21	103.86 ± 14.62	95.53 ± 3.59	89.35 ± 5.79	96.25 ± 4.98
	28	107.85 ± 9.84	93.35 ± 4.68	83.50 ± 5.48	92.59 ± 4.49
	56	115.07 ± 6.09 **	130.79 ± 9.39	104.03 ± 5.96	82.52 ± 2.11
AMI300 + DOX10	7	97.36 ± 10.45	97.82 ± 7.95	105.00 ± 12.85	105.81 ± 9.69
	14	89.73 ± 14.95	98.22 ± 10.08	103.32 ± 7.84	104.59 ± 10.04
	21	91.30 ± 13.75	100.00 ± 2.91	103.67 ± 5.78	101.93 ± 4.56
	28	74.25 ± 14.36 ***^, +++^	106.19 ± 9.15	104.63 ± 5.49	97.52 ± 8.27
	56	90.58 ± 11.38 **^, ++^	104.67 ± 7.67	102.78 ± 6.92	101.53 ± 7.82

Statistical analysis was performed using the Student *t*-test. The results are expressed as a percentage related to the control group on day 0. **, ***—*p* < 0.01, 0.001 for the results in comparison with AMI300. ++, +++—*p* < 0.01, 0.001 for the results in comparison with AMI300 + DOX6. Reference data were set according to Patel et al. [40]. Note: all animals in the DOX10-treated group died 4 weeks after treatment.

## Data Availability

The original contributions presented in this study are included in the article. Further inquiries can be directed to the corresponding authors.

## Abbreviations

The following abbreviations are used in this manuscript:

AKR1A	Aldo-Keto Reductase 1A
ALT	Alanine AminoTransferase
AMI	Amifostine
AST	ASpartate aminoTransferase
ANOVA	The one-way test
BAX	Apoptosis Regulator BAX Protein
BUN	Blood Urea Nitrogen
CAT	Catalase
CBC	Complete Blood Count
CBR1/CBR3	Carbonyl Reductases
COX-2	Cyclooxygenase-2
DNA	DeoxyriboNucleic Acid
DOX	Doxorubicin
HB	Haemoglobin
HCT	Haematocrit
H&E	Haematoxylin & Eosin
HDS	Hepatic Damage Score
H_2_O_2_	Hydrogen Peroxide
8-OHdG	8-hydroxy-2-deoxyguanosine
O_2_^−^	Superoxide Anions
OH^•^	Hydroxyl Radicals
IL-1β	Interleukin-1β
IL-6	Interleukin-6
iNOS	Inducible Nitric Oxide Synthase
MAPK	Mitogen-Activated Protein Kinase
MCH	Mean Corpuscular Haemoglobin
MCHC	Mean Corpuscular Haemoglobin Concentration
MCV	Mean Corpuscular Volume
NF-kB	Nuclear Factor-κB
PLT	Platelets
PMNCs	PolyMorphoNuclear Cells
RBC	Red Blood Cells
RDS	Renal Damage Score
RNA	RiboNucleic Acid
ROS	Reactive Oxygen Species
SCr	Serum Creatinine
SOD	Superoxide Dismutase
TNF-α	Tumour Necrosis Factor-alpha
WBC	White Blood Cells
WR-1065	Free Thiol Metabolite of Amifostine
